# Extremely hard and tough high entropy nitride ceramics

**DOI:** 10.1038/s41598-020-76945-y

**Published:** 2020-11-16

**Authors:** Dmitry Moskovskikh, Stepan Vorotilo, Veronika Buinevich, Alexey Sedegov, Kirill Kuskov, Alexander Khort, Christopher Shuck, Maksim Zhukovskyi, Alexander Mukasyan

**Affiliations:** 1 National University of Science and Technology MISiS, Moscow, 119049 Russia; 2 KTH Royal Institute of Technology, 114 28 Stockholm, Sweden; 3 A.J. Drexel Nanomaterials Institute and Department of Materials Science and Engineering, Drexel University, Philadelphia, PA 19104 USA; 4 Notre Dame Integrated Imaging Facility, University of Notre Dame, Notre Dame, IN 46556 USA; 5 Department of Chemical and Biomolecular Engineering, University of Notre Dame, Notre Dame, IN 46556 USA

**Keywords:** Mechanical properties, Ceramics

## Abstract

Simultaneously hard and tough nitride ceramics open new venues for a variety of advanced applications. To produce such materials, attention is focused on the development of high-entropy ceramics, containing four or more metallic components distributed homogeneously in the metallic sublattice. While the fabrication of bulk high-entropy carbides and borides is well established, high-entropy nitrides have only been produced as thin films. Herein, we report on a newel three-step process to fabricate bulk high-entropy nitrides. The high-entropy nitride phase was obtained by exothermic combustion of mechanically-activated nanostructured metallic precursors in nitrogen and consolidated by spark plasma sintering. The fabricated bulk high-entropy nitride (Hf_0.2_Nb_0.2_Ta_0.2_Ti_0.2_Zr_0.2_)N demonstrates outstanding hardness (up to 33 GPa) and fracture toughness (up to 5.2 MPa∙m^1/2^), significantly surpassing expected values from mixture rules, as well as all other reported binary and high-entropy ceramics and can be used for super-hard coatings, structural materials, optics, and others. The obtained results illustrate the scalable method to produce bulk high-entropy nitrides with the new benchmark properties.

## Introduction

High-entropy (HE) ceramics are solid solutions based on interstitial phases (carbides, borides, silicides, etc.) and contain 4 or more metallic species, which endow them with unique physical and mechanical properties as a result of entropy stabilization^1–3^. HE ceramics have attracted increasing interest, as they surpass binary ceramics (such as TiC, TiN, SiC, Si_3_N_4_, ZrO_2_, Al_2_O_3_) in terms of hardness, fracture toughness, corrosion resistance, and high-temperature stability^4–6^. Among conventional ceramics, transition-metal nitrides (TMN) have historically been used as cutting tools and wear-resistant coatings because of their high hardness and strength, high melting points, excellent thermal conductivity, coupled with thermal and chemical stability^7–10^. The hardness and toughness of TMN and related solid solutions with FCC structure are interrelated functions of electron valence. As the valence electron concentration (VEC) is minimized, the hardness is maximized (usually at VEC = 8.4); oppositely, when the valence is maximized, the toughness is minimized (at VEC ≥ 10)^7,11,12^ as the result of antibonding state occupation-induced lattice softening^13,14^. For example, in δ-TiN_1−x_, the microhardness maximum occurs at δ-TiN_0.67_, which corresponds to 7.3 valence electrons^14^. An increased valence electron concentration resulting from higher nitrogen content leads to a decrease in hardness. This is the reason why zirconium and titanium carbonitrides have lower hardness when carbon atoms are replaced with nitrogen^15^. However, for δ-HfN_1−x_, a smooth increase in microhardness with nitrogen content is observed, due to the difference in 5f electron bonding states^15^. Thus, hardness and fracture toughness are inversely related in nitride ceramics, and bypassing this effect would lead to extraordinary properties and more widespread use.

Ternary nitride (TN) systems are predicted to be supertough—harder and more ductile than binary systems—due to the increased valence electron concentration^12,16,17^. Various research groups have studied the effect of valence electron concentration on mechanical properties for the design of advanced TMNs^17^. According to Balasubramanian et al.^12^ the optimized hardness and toughness in TMNs are expected at VEC of 9.0–9.5. A brittle-to-ductile transition is expected at a critical VEC = 10 and a transition to mechanical instability at VEC = 10.6. However, the calculated phonon dispersion curves indicate a dynamical stability-to-instability transition between VEC = 9 and 10, which is smaller than the critical VEC = 10.6 for the mechanical stability-instability transition. Overall, a narrow region between VEC = 9 and 10 is outlined for the search of phases with the highest toughness. Considering this, Ti_0.5_Ta_0.5_N (VEC = 9.5) possesses one of the highest among TNs theoretical and experimentally measured hardness values up to 31 GPa^17,18^. We believe, this result can be further expanded upon by engineering high-entropy nitrides (HEN).

To date, HENs are predominantly produced as thin films^19–25^, due to their enhanced solubility and phase stability^26^ compared to bulk ceramics. Jin et al.^27^ reported the synthesis of powdered metal HEN V_0.2_Cr_0.2_Nb_0.2_Mo_0.2_Zr_0.2_N_1−x_ by planetary ball milling a mixture of five transition-metal chlorides with urea and subsequent annealing of the reactive mixture under N_2_ flow. The resulting powders were tested as supercapacitors, but neither the sintering nor mechanical testing was reported. Therefore, the goal of our work was the development of fabrication technology and studying the structure, and mechanical properties of a bulk HEN ceramic (Hf_0.2_Nb_0.2_Ta_0.2_Ti_0.2_Zr_0.2_)N.

The fabricated composition was based on the ternary Ti_0.5_Ta_0.5_N ceramic with the highest reported hardness^17,18,28,29^. Three additional metallic constituents were added to produce the entropy stabilization effect^3^. The additional metals were chosen to retain VEC close to 9.5. The article shows the results of the successful application of the complex technology of CS-SPS consolidation of mechanically activated powders for bulk HEN ceramics production and can be used for mass production of high-quality bulk HE ceramics of different types.

## Results and discussion

The convex hulls for the Hf–Nb–Ta–Ti–Zr–N system were constructed at various chemical nitrogen potentials (μ_N_) to estimate the range where all constituent metals could form mono-nitrides (Supplementary Table S-I in Supplementary Information). The decrease of μ_N_ corresponds to an increase in temperature or decrease of nitrogen partial pressure in the system. The stability range of mono-nitrides decreases in the following order: TiN (space group Fm$\stackrel{-}{3}$m, stable at − 7.952 < μ_N_ < − 11.249), ZrN (space group Fm$\stackrel{-}{3}$m, stable at − 8.584 < μ_N_ < − 11.399), HfN (space group Fm$\stackrel{-}{3}$m, stable at − 9.501 < μ_N_ < − 11.463), TaN (space group P$\stackrel{-}{6}$2m, stable at − 9.033 < μ_N_ < − 10.98), NbN (space group P$\stackrel{-}{6}$m2, stable at − 8.778 < μ_N_ < − 9.98). The μ_N_ range in which all five mono-nitrides coexist is limited to − 9.033 < μ_N_ < − 9.98. This limited stability range complicates the synthesis of bulk HENs and accounts for the lack of published reports on this topic.

The MaterialsProject database contains the data on multiple TNs: Zr_0.5_Ti_0.5_N, Hf_0.5_Zr_0.5_N, Ta_0.5_Nb_0.5_N, Ta_0.5_Ti_0.5_N, Zr_0.5_Nb_0.5_N, Ti_0.5_Nb_0.5_N, Hf_0.5_Ti_0.5_N, TaTi_2_N_3_, etc. Among them, only one TN phase Hf_0.5_Zr_0.5_N (space group R$\stackrel{-}{3}$m) was found to be stable in the investigated system (stability range − 9.333 < μ_N_ < − 11.492). Although its energy of formation from HfN and ZrN is relatively low (− 5 kJ/mol at 0 °C), the relatively broad stability range increases the probability of formation of Hf_0.5_Zr_0.5_N as an intermediate phase during the employed three-step processing. This finding is consistent with the predictions of Sun et.al.^30^, who indicated HfZrN as the only stable TN in the TM1-TM2-N systems (TM = Ta, Hf, Ti, Zr, Nb, V).

Most of the binary nitrides in TM1-TM2-N systems (TM = Ti, Zr, Hf, Ta, Nb, V) are predicted to be thermodynamically unstable yet meta-stabilizable^30^. In a narrow μ_N_ interval of − 9.333 < μ_N_ < − 9.033, the Hf_0.5_Zr_0.5_N phase undergoes decomposition while all 5 mono-nitrides retain stability (Supplementary Table S-I). The co-existence of mono-nitride phases might be instrumental for the formation of a HEN solid solution.

**Figure 1:**
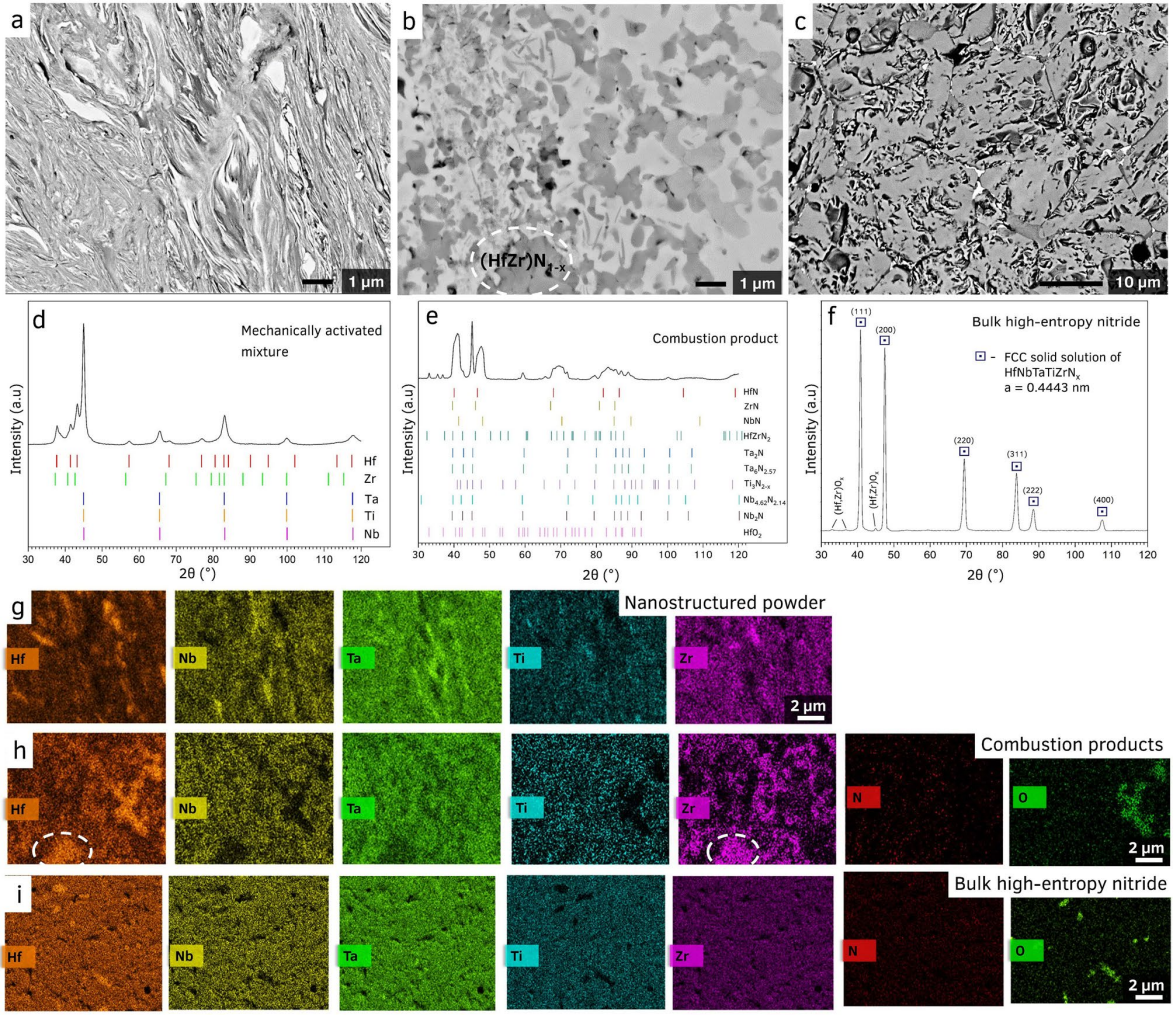
SEM cross-sections of (**a**) mechanically activated mixture Hf + Nb + Ta + Ti + Zr, (**b**) combustion products, (**c**) and spark plasma sintered high-entropy nitride. Corresponding XRD patterns of the (**d**) mechanically activated mixture Hf + Nb + Ta + Ti + Zr mixture, (**e**) after combustion synthesis, and (**f**) after spark plasma sintering. The EDS maps for the (**g**) mechanically activated mixture show that the phases have close contact, but are still separated, (**h**) combustion products showing multiple distinct elemental distributions, and (**i**) showing the fully compact, homogeneous HEN.

The first preparation stage allowed us to make composite particles involving all five metals mixed on sub-micron sized layers of metallic constituents (Fig. 1a,g). Due to the cold-welding phenomenon during milling, thin layers of metals with a thickness of 10–100 nm formed in the bulk of each particle, providing high homogeneity for their mixing. The EDS analysis indicated that the ratio between the metals remained to be equimolar. Also, milling at relatively low speed (200 rpm) and under high pressure of pure argon allowed to produce powders with low concentrations of impurities. The XRD patterns (Fig. 1d) of thus prepared powders showed that all metals retained their crystallinity even after 10 h of mechanical treatment.

The goal of the second preparation stage is to introduce nitrogen into the system in the form of metal nitrides, as well as nitrogen solid solutions. This task was accomplished by using energy-saving combustion synthesis method^31^. Supplementary Table S-II demonstrates that the reactions between considered metals and nitrogen are highly exothermic with adiabatic combustion temperature well above 3000 K. The reaction between the metal composite particles and nitrogen was initiated locally by hot tungsten wire, followed by the rapid combustion front propagation along with the media. The total nitridation process duration was ~ 5 s. It can be seen that the combustion of such reactive metal particles in a nitrogen atmosphere leads to the formation of multiple nitride phases (Fig. 1b,e), among which there was detected the predicted Hf_0.5_Zr_0.5_N_1-x_ TN phase. The microstructure of the inner part of the composite particles became coarser (Fig. 1b) with the grain size in the range 0.5–1 μm. A mixed hafnium-zirconium-based oxide phase was also present in the combustion products (Fig. 1h), which presumably originated due to the oxygen impurities in the initial powders and exposure of mechanically activated mixture to air during the pressing of green pellets for combustion synthesis.

In the third stage, the SPS of the synthesized complex metal nitride particles were used to produce bulk ceramics. After the SPS at the experimentally optimized conditions, the measured relative density of the ceramics was 96.6% of the theoretical maximum. The obtained HEN phase is characterized by narrow grain size distribution (10–16 μm) and crystals of polyhedric, mostly hexagonal, shape (Fig. 1c). Moreover, the Hf_0.5_Zr_0.5_N_1−x_ TN phase was not found in the sintered specimens (Fig. 1f,i), indicating its successful conversion into the HEN. The lattice constant *a* = 0.4443 was calculated based on the XRD pattern of the HEN (Fig. 1f). According to the results of XRD and EDS analysis ~ 4.2 mol% of the (Hf,Zr)O_x_ phase, formed during the combustion synthesis, retained in the sintered ceramics (Fig. 1f,i). The amount of oxides is relatively small and, we suppose, could be removed by applying additional technological steps or by adjusting the technological process.

**Figure 2:**
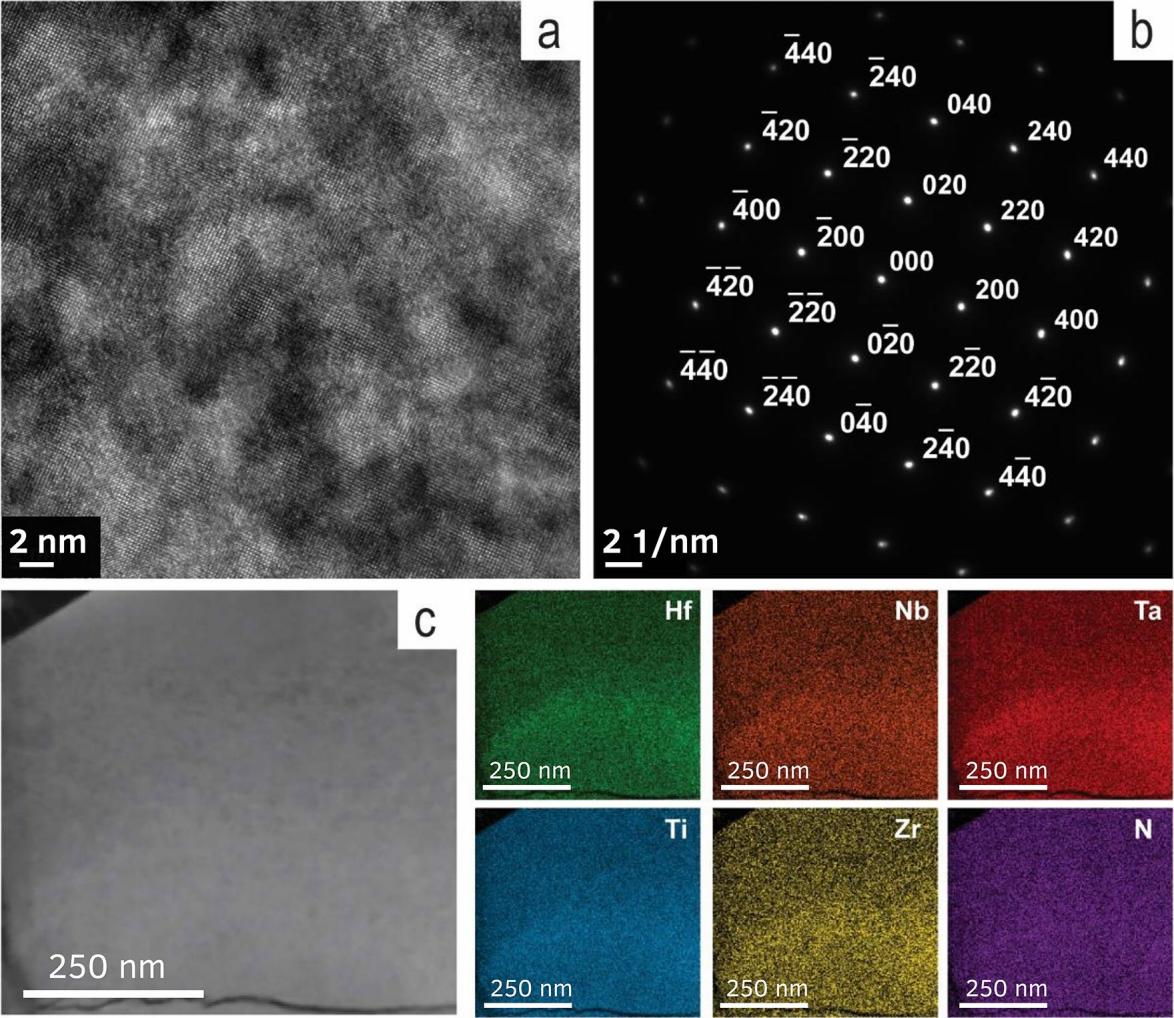
(**a**) HRTEM image of spark plasma sintered high-entropy nitride; (**b**) selected area electron diffraction of (HfNbTaTiZr)N_x_ taken along the [001] zone axis; (**c**) EDS elemental mapping of the (HfNbTaTiZr)N_x_ phase.

Detailed TEM investigations (Fig. 2) were performed to confirm the presence of a HEN in the sintered specimens and to define the composition of the HEN phase. HRTEM (Fig. 2a) and selected area diffraction pattern (SAED) (Fig. 2b) confirm the single-phase structure of sintered high entropy nitride. The indexed SAED pattern revealed the FCC structure of HEN with the d-spacing values d_020_ = 0.221 nm, d_200_ = 0.221 nm, d_220_ = 0.157 nm, and d_240_ = 0.099 nm. The value of SAED-derived lattice constant *a* = 0.443 ± 0.001 nm is in excellent agreement with the value calculated from XRD data. Advanced EDS mapping (Fig. 2c) demonstrates the uniform distributions of all elements along with the HEN phase. The EDS analysis of different selected areas (Supplementary Table S-III) showed the following statistically proven composition of the phase in at. %: N (28.9 ± 3.2); Hf (11.0 ± 0.4), Zr (11.0 ± 0.8); Ti (15.1 ± 0.5); Nb (15.8 ± 0.8) and Ta (18.0 ± 0.9). The lower amount of Hf and Zr in comparison with other metals could be explained by the formation of crystals of separate (Hf,Zr)O_x_ phase during the CS step and its recrystallization and consolidation during the SPS (Fig. 1f and Supplementary Fig. S1).

**Figure 3:**
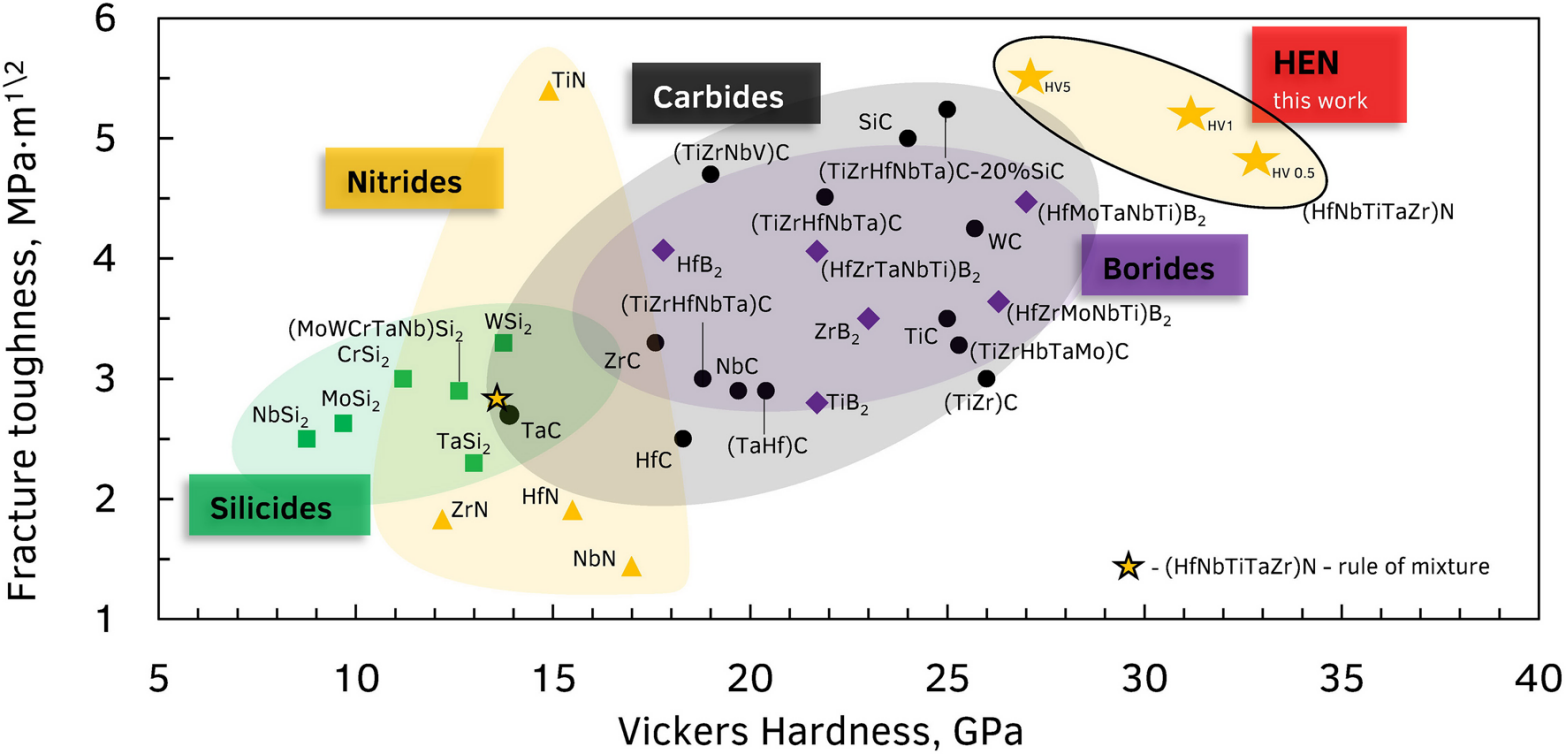
Fracture toughness versus hardness plot with measured values for high-entropy nitride and previously reported ceramics.

The hardness and Vickers fracture toughness of this high entropy nitride were measured at 4.9–98 N loads and compared to mono-nitrides and nitride solid solutions reported in the literature (Supplementary Table S-IV). The measured hardness HV_0.5_ and elastic modulus of HEN are 32.8 ± 1.6 GPa and 352 ± 17 GPa, respectively. The elastic modulus value is comparable with the value of 360 GPa, obtained in coatings^32^. An increase in the indentation load leads to a gradual decrease of the hardness to HV_10_ = 22.5 ± 1.4 GPa (Fig. 3). However, the experimentally measured values of hardness and fracture toughness of the HEN ceramic surpasses the values calculated based on the rule of the mixture on 130% and 82%, respectively (HV_1_ = 31.2 ± 3.6 GPa and K_1C_ = 5.2 ± 0.18 MPa are the experimental data, and m^1/2^ROM HV_1_ = 13.6 GPa, K_1C_ = 2.85 MPa∙m^1/2^ are the calculated). It should be noticed, the measured results for our HEN are significantly higher the previously reported values for carbide, nitride, and silicide ceramics, including the HE compositions (Fig. 3). Moreover, the hardness values of HEN (Hf_0.2_Nb_0.2_Ta_0.2_Ti_0.2_Zr_0.2_)N far surpassed the estimations for both TNs phases Ti_0.5_Nb_0.5_N (17.3 GPa) and Ti_0.5_Ta_0.5_N (17.5 GPa) by Sangiovanni^17^. The breakdown of the values provided in Fig. 3 can be found in Supplementary Information (Supplementary Table S-IV).

Earlier, Sarker et al.^3^ described an explicit strengthening effect in HE carbides. The hardness of sintered Hf_0.2_Nb_0.2_Ta_0.2_Ti_0.2_Zr_0.2_C ceramic of 32 GPa is 40% above the value calculated by the rule of mixture (23 GPa). A similar entropy-induced strengthening effect might be responsible for the increased hardness of the HEN, obtained in this work. Previously, enhancement of the mechanical performance of TMN was achieved by engineering alternating layers of TMNs and more ductile body-centered cubic metals^33^. Similarly, nanocomposite structures developed by Voevodin and Zabinski^34^ demonstrates high hardness at stresses below the elastic strength limit, while at extreme loading their mechanical behavior switches to ductile, thus preventing brittle failure. A related effect might be responsible for the increase of the fracture hardness of the HENs due to possible nanoscale precipitates of ductile elements (i.e. Ta) on the boundaries of HEN grains during the sintering.

Moreover, as the valence electron concentration of the HENs is close to the optimal value of 9.5, derived by Guo^35^ and Sangiovanni^17^, the “lattice softening” effect could also contribute to the increased fracture toughness of the HEN phase. The effect of a simultaneous considerable increase of hardness and fracture toughness in HEN warrants closer investigation and theoretical modeling. However, these results indicate that HENs have the potential to become the new benchmark ceramic for structural and machining applications since the mechanical performance of the (HfNbTaTiZr)N ceramic developed in this work is considerably superior to conventional SiC, TiC, TiN, and TiB_2_.

## Conclusions

1. Grand potential phase diagram modeling revealed that mono-nitrides of Hf, Zr, Ta, Nb, and Ti are stable at relatively narrow nitrogen potential range − 9.033 < μ_N_ < − 9.98 and that trigonal Hf_0.5_Zr_0.5_N is the only stable at − 9.333 < μ_N_ < − 11.492 TN in the system. A nitrogen potential range was indicated (− 9.033 < μ_N_ < − 9.333) where this trigonal phase will decompose into FCC ZrN and HfN to facilitate the formation of FCC HEN solid solution.
2. Based on the proposed model, a three-stage synthesis protocol was developed to produce bulk HEN ceramics, including mechanical treatment of the metallic constituents in an argon atmosphere, combustion of mechanically-induced nanostructured particles in nitrogen, and spark plasma sintering of the combustion products.
3. The bulk ceramics are primarily composed of hexagonal grains (10–15 μm) of the HEN phase. The sintered HEN (Hf_0.2_Nb_0.2_Ta_0.2_Ti_0.2_Zr_0.2_)N demonstrates outstanding hardness (up to 33 GPa) and fracture toughness (5.2 MPa∙m^1/2^), which is significantly higher in comparison with theoretical estimations and carbide, nitride, and silicide ceramics, including TNs and other HEs.
4. The strengthening effect presumably results from entropy stabilization and optimization of valence electron concentration of the HEN phase. The obtained ceramic is a promising candidate for practical application in multiply areas, where special mechanical properties are required.
5. A scalable approach for the fabrication of bulk HEN by the complex CS-SPS consolidation of mechanically activated powders, is proposed for the first time. While the only one HEN system was studied here, countless possible other HENs can be produced using the developed technological approach.

## Methods

### Calculation of grand potential phase equilibria

To analyze the phase equilibria in this system, the formation enthalpies for nitride phases were calculated using mixed GGA and GGA + U (semiempirically-tuned generalized gradient approximations) frameworks, which is known for its ability to correctly predict the phase stability^36^. Grand potential phase diagrams at varied nitrogen potentials were calculated using PDApp software, which is integrated into Materials API^37,38^ and employs a database of DFT computed bulk material energies with crystal structures obtained from the Inorganic Crystal Structure Database (ICSD)^39^ and those generated by applying data-mined chemical substitutions^40,41^.

### Fabrication of high-entropy ceramics

**Figure 4:**
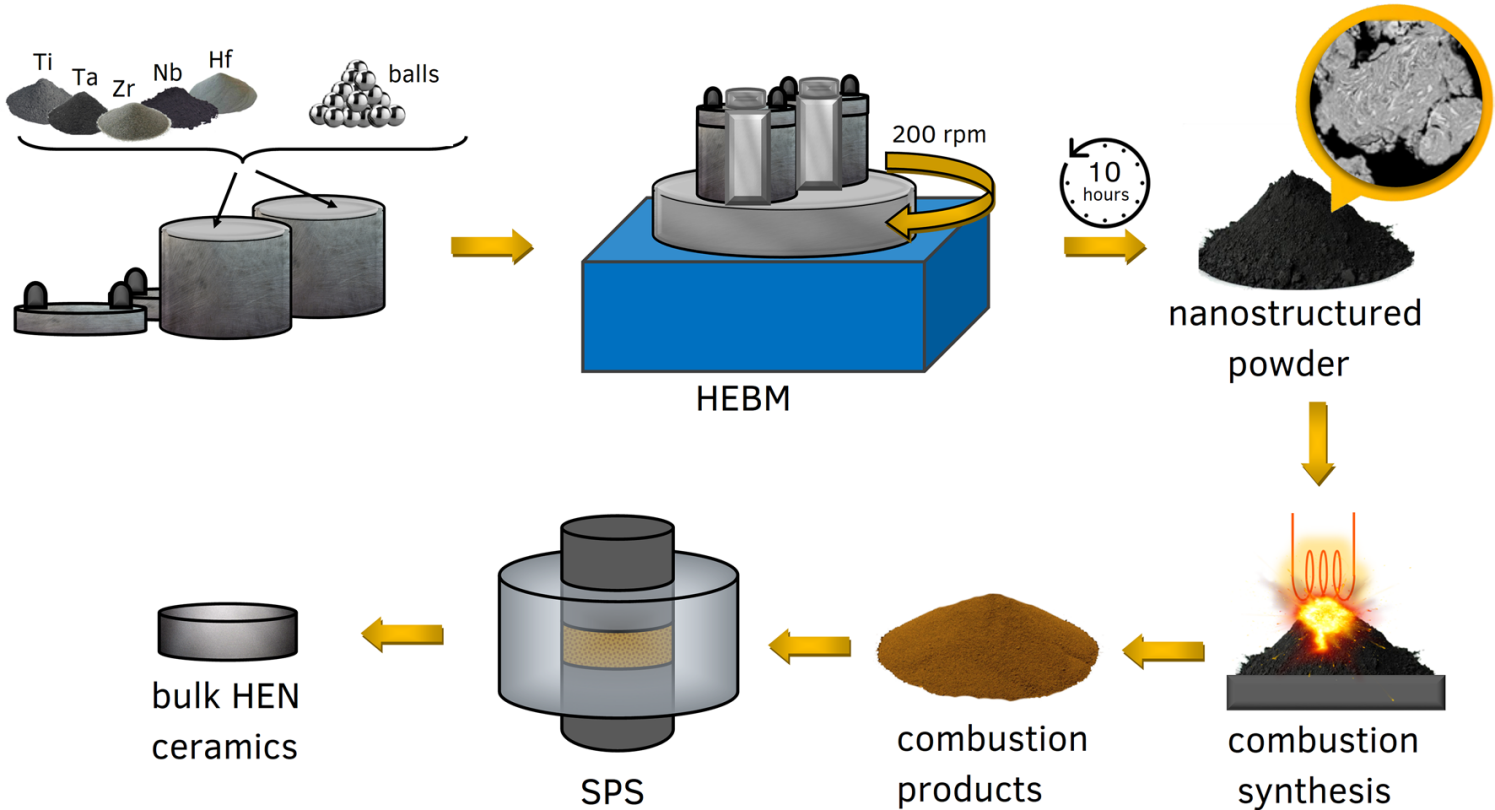
Schematic showing the fabrication process. Initially, the metal powders are HEBM in a planetary ball mill for 10 h. This mixture then undergoes CS under a nitrogen atmosphere. Finally, the powders are transferred to an SPS device, where they undergo the final stage of reaction/consolidation in a nitrogen atmosphere.

Figure 4 provides the schematic for the three-stage process employed for the synthesis of bulk high-entropy nitride ceramics. The overall processing method includes three stages: (i) preparation of the reactive nanocomposite powders by high energy ball milling (HEBM); (ii) combustion synthesis (CS) of TNs; and (iii) spark plasma sintering (SPS) of the bulk HEN ceramics.

### Preparation of reactive composite powders

The metallic powders of Hf, Nb, Ta, Ti and Zr (RusRedMet, Russia, > 99% purity) with a particle size distribution of 40–60 µm were used for preparation of a precursor mixtures. HEBM of reactive mixtures was conducted in an argon atmosphere (4 atm, 99.998%) using a double-station planetary ball mill (Activator-2s, Russia) equipped with steel mill and steel grinding medium (balls). Batches consisted of 20 g powders and were mixed in an equimolar ratio Hf:Nb:Ta:Ti:Zr = 1:1:1:1:1 using 250 ml steel jars and 6 mm steel balls. The ball to powder mixture weight ratio was 20:1, The milling speed was 200 rpm at a rotational coefficient of K = 1. The total duration of the mechanical treatment was 10 h. The goal is to produce nanostructured composite particles, which involve all five components.

### Combustion synthesis

Mechanically induced nanostructured composite Hf/Nb/Ta/Ni/Zr particles were placed in a laboratory chemical reactor. Initially, the reactor was vacuumed and then filled with gaseous nitrogen up to 8 atm. Powder mixtures were locally preheated using a hot tungsten wire to initiate a chemical reaction with the subsequent propagation of a self-sustaining combustion front. At this stage, the metal nitrides were synthesized. The combustion products were then ball-milled for 2 h at 60 rpm using WiseMixSBML mill (DAIHAN Scientific, South Korea) equipped with 250 ml steel jars and 6 mm steel balls. The ball to powder mixture weight ratio was 6:1.

### Spark Plasma Sintering of bulk ceramics

The synthesized powders were consolidated in an SPS system (Labox 650, SinterLand, Japan) in a nitrogen atmosphere (0.8 atm) at 2073 K. The dwell time was 20 min at a pressure of 30 MPa, the heating rate was 100 °C/min. Bulk ceramic samples in the form of disks with a diameter of 20 mm and a thickness of 5–6 mm were produced by SPS.

### Material characterization

X-ray diffraction (XRD) was applied for the study of phase composition of the fabricated materials using DRON-4-07 (Russia) monochromatic Co-Kα radiation. The structure of the experimental materials was analyzed via scanning electron microscopy (SEM) on a Vega 3 (TESCAN, Czech Republic) and JSM-7600F (JEOL, Japan) with a microanalysis system (EDX, Oxford Instruments) and a high-resolution transmission electron microscopy (TEM) on TITAN 800-300 (Thermo Fisher Scientific, USA) equipped with an Ultim Max EDS system (Oxford Instruments).

Vickers hardness tests were used for the study of the microhardness of the synthesized materials [Emco-Test DuraScan 70 (Austria)]. The applied loads varied from 0.5 to 10 N. The fracture toughness was measured using the Vickers indentation-induced cracks corresponding to the Anstis method^42^. The elastic modulus was measured by Anton Paar CSM Micro Indentation Tester (Austria) under applied loads of 100 mN.

## Supplementary information

Supplementary Information.
