# Supplementary material for: Extremely hard and tough high entropy nitride ceramics
Source: Sci Rep. 2020 Nov 16;10:19874. doi: 10.1038/s41598-020-76945-y (PMC7669861; doi:10.1038/s41598-020-76945-y)
Supplement: Supplementary file 1 — Supplementary Information. [file 41598_2020_76945_MOESM1_ESM.docx]

**SUPPLEMENTARY MATERIALS**

**Extremely hard and tough high entropy nitride ceramics**

Dmitry Moskovskikh^1^, Stepan Vorotilo^1^, Veronika Buinevich^1^, Alexey Sedegov^1^, Kirill Kuskov^1^, Alexander Khort^2^, Christopher Shuck^3^, Maksim Zhukovskyi^4^, Alexander Mukasyan^5^

*^1^ National University of Science and Technology MISiS, Moscow 119049, Russia*

*^2^KTH Royal Institute of Technology, Stockholm 114 28, Sweden*

*^3^ A.J. Drexel Nanomaterials Institute and Department of Materials Science and Engineering, Drexel University, Philadelphia, PA 19104, USA*

*^4^ Notre Dame Integrated Imaging Facility, University of Notre Dame, Notre Dame, IN, 46556 USA*

*^5^ Department of Chemical and Biomolecular Engineering, University of Notre Dame, Notre Dame, IN, 46556 USA*

Correspondence and requests for materials should be addressed to A.K. (email: [khort@kth.se](mailto:khort@kth.se)) and D.M. (email: mos@misis.ru)

| **μN, eV** | **Convex hull phases (Formation/decomposition energy = 0 eV/atom)** | | | | |
| --- | --- | --- | --- | --- | --- |
|  | **Ti-based** | **Hf-based** | **Ta-based** | **Nb-based** | **Zr-based** |
| -8.584<μ_N_<-7.952 | TiN  (Fm$\bar{3}$m) | Hf_3_N_4_  (Pnma) | Ta_3_N_5_  (Cmcm) | Nb_5_N_6_  (P$\bar{6}$_3_mсm) | Zr_3_N_4_  (Pnma) |
| -8.778<μ_N_<-8.584 |  |  |  |  | ZrN  (Fm$\bar{3}$m) |
| -8.994<μ_N_<-8.778 |  |  |  | NbN  (P$\bar{6}$m2) |  |
| -9.033<μ_N_<-8.994 |  |  | Ta_5_N_6_  (P6_3_/mcm) |  |  |
| -9.501<μ_N_<-9.033 |  |  | TaN  (P$\bar{6}$2m) |  |  |
| -9.98<μ_N_<-9.501 |  | HfN  (Fm$\bar{3}$m) |  |  |  |
| -10.277<μ_N_<-9.98 |  |  |  | Nb_2_N  (P$\bar{3}1$m) |  |
| -10.937<μ_N_<-10.277 |  |  | Ta_2_N  (P$\bar{3}1$m) |  |  |
| -11.087<μ_N_<-10.937 |  |  |  | Nb  (Im$\bar{3}$m) |  |
| -11.249<μ_N_<-11.087 |  |  | Ta  (Im$\bar{3}$m) |  |  |
| -11.399<μ_N_<-11.249 | Ti_2_N  (P4_2_/mnm) |  |  |  |  |
| -11.463<μ_N_<-11.399 |  |  |  |  | Zr_2_N  (P4_2_/mnm) |
| -11.822<μ_N_<-11.463 |  | Hf_3_N_2_  (R$\bar{3}$m) |  |  |  |
| -11.987<μ_N_<-11.822 |  | Hf_2_N  (P4_2_/mnm) |  |  |  |
| -12.083<μ_N_<-11.987 |  |  |  |  | Zr  (P6_3_/mmc) |
| -12.287<μ_N_<-12.083 |  | Hf  (P6_3_/mmc) |  |  |  |
| -∞<μ_N_<-12.287 | Ti  (P6/mmm) |  |  |  |  |

**Table S-I.** Convex hull phases for the Hf-Ta-Ti-Nb-Zr-N system at varied nitrogen potential.

| **System** | **Adiabatic combustion temperature, K** |
| --- | --- |
| HfN | 5100 |
| ZrN | 4900 |
| TaN | 3360 |
| TiN | 4900 |
| NbN | 3322 |

**Table S-II.** Combustion temperature of the reactions between considered metals and nitrogen ^1^.

| **Element** | **Area1, At.%** | **Area2, At.%** | **Area3, At.%** | **Area4, At.%** | **Area4, At.%** | **Average, At.%** |
| --- | --- | --- | --- | --- | --- | --- |
| N | 28.3 | 25 | 28.7 | 28.9 | 33.8 | 28.9 ± 3.2 |
| Ta | 18.9 | 19.2 | 18.1 | 17.9 | 16.9 | 18.2 ± 0.9 |
| Nb | 16.0 | 16.4 | 16.2 | 15.9 | 14.4 | 15.8 ± 0.8 |
| Ti | 15.3 | 15.7 | 15.1 | 15.2 | 14.3 | 15.1 ± 0.5 |
| Hf | 11.2 | 11.4 | 10.8 | 11.2 | 10.4 | 11.0 ± 0.4 |
| Zr | 10.3 | 12.3 | 11.1 | 10.9 | 10.2 | 11.0 ± 0.8 |

**Table S-III**. EDS derived atomic composition obtained from 5 different locations along with calculated average composition.

| **Materials** | **Vickers hardness, HV (GPa)** | **Fracture toughness, MPa∙m^1/2^** |
| --- | --- | --- |
| HfZrTaNbTi)N - exp | 98N (HV_10_) 22.5 ± 1.4  49N (HV_5_) 26.5 ± 1.9  29.4N (HV_3_) 27.1 ± 1.9  9.8N (HV_1_) 31.2 ± 3.6  4.9N (HV_0.5_) 32.8 ± 1.6 | 5.0 ± 0.15  4.8 ± 0.21  5.2 ± 0.18 |
| HfZrTaNbTi)N – rule of mixture | HV_1_=13.6 | 2.85 |
| HfN | HV_1_ = 15.5 ^2^ | 1.91 ^3^ - calc |
| ZrN | HV_1_ = 12.2 ^2^ | 1.83 ^3^ - calc |
| NbN | HV_1_ = 17.0 ^2^ | 1.44 ^3^ - calc |
| TiN | HV_20_ = 14.9 ^4^ | 5.4 ^5^ |
| Ti_0.5_Nb_0.5_N | 17.3 ^6^ - calc | - |
| Ti_0.5_Ta_0.5_N | 17.5^6^ - calc | - |
| HfC | 18.3 | 2.5 ^7^ |
| WC | 25.71 | 4.54 ^8^ |
| ZrC | 17.6 | 3.30 ^9^ |
| TaC | 13.9 | 2.7 ^10^ |
| NbC | 19.7 | 2.9 ^11^ |
| TiC | 28.6 | 3.9 ^12^ |
| SiC | 24 | 5 ^13^ |
| TiC–ZrC | 26 | 3.0 ^14^ |
| TaC–HfC | 20.4 | 2.9 ^10^ |
| (TiZrHfNbTa)C | 21.9 ± 0.4 | 4.51 ± 0.61 ^15^ |
| (TiZrHfNbTa)C-20%SiC | 25.0 ± 0.8 | 5.24 ± 0.41 ^15^ |
| (TiZrNbTaMo)C | 25.3 ± 0.3 (9.8 N) | 3.28 ± 0.12 ^16^ |
| (TiZrNbTaHf)C | 18.8 (9.8 N) | 3.0 ^17^ |
| (TiZrNbV)C | 19 (49 N) | 4.7 ^18^ |
| ZrB_2_ | 23 | 3.5 ^19^ |
| HfB_2_ | 17.8 | 4.07 ^20^ |
| TiB_2_ | 21.7 | 2.8 ^21^ |
| (HfZrTaNbTi)B_2_ | 21.7 | 4.06 ^22^ |
| (HfZrMoNbTi)B_2_ | 26.3 | 3.64 ^22^ |
| (HfMoTaNbTi)B_2_ | 27.0 | 4.47 ^22^ |
| (MoWCrTaNb)Si_2_ | 12.62 | 2.9 ^23^ |
| WSi_2_ | 13.75 | 3.3 ^23^ |
| MoSi_2_ | 9.69 | 2.63 ^24^ |
| CrSi_2_ | 11.2 | 3 ^25^ |
| TaSi_2_ | 13 | 2.3 ^26^ |
| NbSi_2_ | 8.76 | 2.5 ^27^ |

**Table S-IV.** Vickers hardness and fracture toughness of different ceramics.


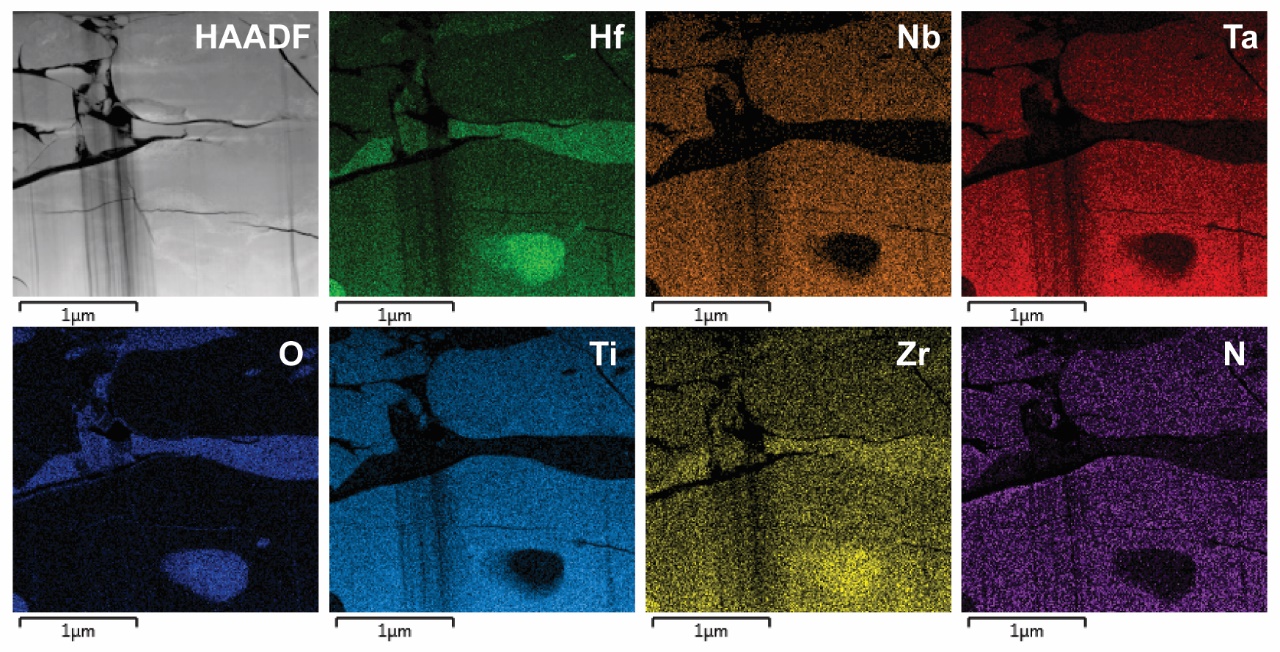


**Figure S1.** Low resolution EDS maps of spark plasma sintered high-entropy nitride.

# References

1. Yi, H. C. & Moore, J. J. Self-propagating high-temperature (combustion) synthesis (SHS) of powder-compacted materials. *J. Mater. Sci.* **25**, 1159–1168 (1990).

2. Chen, X.-J. *et al.* Hard superconducting nitrides. *Proc. Natl. Acad. Sci.* **102**, 3198–3201 (2005).

3. Mei, Z.-G., Bhattacharya, S. & Yacout, A. M. First-principles study of fracture toughness enhancement in transition metal nitrides. *Surf. Coatings Technol.* **357**, 903–909 (2019).

4. Delbari, S. A., Nayebi, B., Ghasali, E., Shokouhimehr, M. & Shahedi Asl, M. Spark plasma sintering of TiN ceramics codoped with SiC and CNT. *Ceram. Int.* **45**, 3207–3216 (2019).

5. Lee, C.-H., Lu, H.-H., Wang, C.-A., Nayak, P. K. & Huang, J.-L. Microstructure and mechanical properties of TiN/Si3N4 nanocomposites by spark plasma sintering (SPS). *J. Alloys Compd.* **508**, 540–545 (2010).

6. Sangiovanni, D. G., Hultman, L. & Chirita, V. Supertoughening in B1 transition metal nitride alloys by increased valence electron concentration. *Acta Mater.* **59**, 2121–2134 (2011).

7. Sciti, D., Guicciardi, S. & Nygren, M. Densification and Mechanical Behavior of HfC and HfB 2 Fabricated by Spark Plasma Sintering. *J. Am. Ceram. Soc.* **91**, 1433–1440 (2008).

8. Zheng, D., Li, X., Ai, X., Yang, C. & Li, Y. Bulk WC–Al2O3 composites prepared by spark plasma sintering. *Int. J. Refract. Met. Hard Mater.* **30**, 51–56 (2012).

9. Acicbe, R. B. & Goller, G. Densification behavior and mechanical properties of spark plasma-sintered ZrC–TiC and ZrC–TiC–CNT composites. *J. Mater. Sci.* **48**, 2388–2393 (2013).

10. Cedillos-Barraza, O. *et al.* Sintering behaviour, solid solution formation and characterisation of TaC, HfC and TaC–HfC fabricated by spark plasma sintering. *J. Eur. Ceram. Soc.* **36**, 1539–1548 (2016).

11. Demirskyi, D., Sakka, Y. & Vasylkiv, O. High-temperature reactive spark plasma consolidation of TiB2–NbC ceramic composites. *Ceram. Int.* **41**, 10828–10834 (2015).

12. Sabahi Namini, A., Ahmadi, Z., Babapoor, A., Shokouhimehr, M. & Shahedi Asl, M. Microstructure and thermomechanical characteristics of spark plasma sintered TiC ceramics doped with nano-sized WC. *Ceram. Int.* **45**, 2153–2160 (2019).

13. Moskovskikh, D. O., Song, Y., Rouvimov, S., Rogachev, A. S. & Mukasyan, A. S. Silicon carbide ceramics: Mechanical activation, combustion and spark plasma sintering. *Ceram. Int.* **42**, 12686–12693 (2016).

14. Li, Y., Katsui, H. & Goto, T. Effect of heat treatment on the decomposition of TiC–ZrC solid solutions by spark plasma sintering. *J. Eur. Ceram. Soc.* **36**, 3795–3800 (2016).

15. Lu, K. *et al.* Microstructures and mechanical properties of high-entropy (Ti0.2Zr0.2Hf0.2Nb0.2Ta0.2)C ceramics with the addition of SiC secondary phase. *J. Eur. Ceram. Soc.* **40**, 1839–1847 (2020).

16. Wang, K. *et al.* Microstructure and mechanical properties of (TiZrNbTaMo)C high-entropy ceramic. *J. Mater. Sci. Technol.* **39**, 99–105 (2020).

17. Ye, B., Wen, T., Huang, K., Wang, C. & Chu, Y. First‐principles study, fabrication, and characterization of (Hf 0.2 Zr 0.2 Ta 0.2 Nb 0.2 Ti 0.2 )C high‐entropy ceramic. *J. Am. Ceram. Soc.* **102**, 4344–4352 (2019).

18. Ye, B. *et al.* First-principles study, fabrication and characterization of (Zr0.25Nb0.25Ti0.25V0.25)C high-entropy ceramics. *Acta Mater.* **170**, 15–23 (2019).

19. Chamberlain, A. L., Fahrenholtz, W. G., Hilmas, G. E. & Ellerby, D. T. High-Strength Zirconium Diboride-Based Ceramics. *J. Am. Ceram. Soc.* **87**, 1170–1172 (2004).

20. Ni, D.-W., Zhang, G.-J., Kan, Y.-M. & Wang, P.-L. Hot Pressed HfB 2 and HfB 2 -20 vol%SiC Ceramics Based on HfB 2 Powder Synthesized by Borothermal Reduction of HfO 2 *. *Int. J. Appl. Ceram. Technol.* **7**, 830–836 (2010).

21. Bhaumik, S. K., Divakar, C., Singh, A. K. & Upadhyaya, G. S. Synthesis and sintering of TiB2 and TiB2–TiC composite under high pressure. *Mater. Sci. Eng. A* **279**, 275–281 (2000).

22. Zhang, Y. *et al.* Microstructure and mechanical properties of high-entropy borides derived from boro/carbothermal reduction. *J. Eur. Ceram. Soc.* **39**, 3920–3924 (2019).

23. Liu, D., Huang, Y., Liu, L. & Zhang, L. A novel of MSi2 high-entropy silicide: Be expected to improve mechanical properties of MoSi2. *Mater. Lett.* **268**, 127629 (2020).

24. Chen, F., Xu, J., Liu, Y. & Cai, L. In situ reactive spark plasma sintering of WSi2/MoSi2 composites. *Ceram. Int.* **42**, 11165–11169 (2016).

25. Fischer, D. S. & Schuh, C. A. Microstructure and fracture of anomalous eutectic silicon-disilicide composites. *Intermetallics* **19**, 1661–1673 (2011).

26. Park, H.-K. *et al.* Simultaneous synthesis and consolidation of nanostructured TaSi2–Si3N4 composite by pulsed current activated combustion. *Ceram. Int.* **35**, 99–104 (2009).

27. Ko, I.-Y. *et al.* Pulsed current activated combustion synthesis and consolidation of ultrafine NbSi2 from mechanically activated powders. *Met. Mater. Int.* **15**, 399–403 (2009).
